# A genetic variation of the p38β promoter region is correlated with an increased risk of sporadic colorectal cancer

**DOI:** 10.3892/ol.2013.1334

**Published:** 2013-05-08

**Authors:** QINGHUA HUANG, DIANKE CHEN, SHUNXIN SONG, XINHUI FU, YISHENG WEI, JIACHUN LU, LEI WANG, JIANPING WANG

**Affiliations:** 1 Department of Colorectal Surgery, The Sixth Affiliated Hospital of Sun Yat-Sen University, Guangzhou, Guangdong 510655;; 2 Gastrointestinal Institute of Sun Yat-Sen University, The Sixth Affiliated Hospital of Sun Yat-Sen University, Guangzhou, Guangdong 510655;; 3 Department of Gastrointestinal Surgery, The Second Affiliated Hospital of Guangzhou Medical College, Guangzhou, Guangdong 510620;; 4 The Institute for Chemical Carcinogenesis, The State Key Lab of Respiratory Disease, Guangzhou Medical College, Guangzhou, Guangdong 510182, P.R. China

**Keywords:** case-control study, colorectal cancer, p38β, single nucleotide polymorphisms

## Abstract

p38 plays a critical role in the proliferation, survival, migration and metastasis of colorectal cancer (CRC) cells. The present study assessed the correlation between a single nucleotide polymorphism (SNP) in the p38β promoter region (rs2235356, -1628A>G) and the predisposition of individuals to sporadic CRC in a case-control study. A genotyping method was developed to detect this SNP, using polymerase chain reaction-restriction fragment length polymorphism (PCR-RFLP) analysis. A logistic regression analysis was used to determine the odds ratio (OR) and 95% confidence interval (CI). It was revealed that the -1628G variant allele was correlated with an increased risk of CRC (OR, 1.99; 95% CI, 1.60–2.47; P<0.0001). An *in silico* analysis revealed several transcription factors that either acquired or lost the ability to bind to -1628AA in the p38β promoter region due to the SNP. Therefore, this allelic variant may be a genetic modifier for CRC susceptibility.

## Introduction

Colorectal cancer (CRC) is the third most frequently diagnosed type of cancer worldwide, accounting for >1 million cases and 639,000 mortalities every year (1). The incidence of CRC is currently markedly increasing in developing countries, including China (2). In a recent study, CRC was ranked in the top five causes of mortality in urban and rural areas (9.78 and 5.96 fatalities per 100,000, respectively) of China (3). CRC is a heterogeneous disease affected by genetic and environmental factors (4). It is widely accepted that the mutation of the adenomatous polyposis coli (*Apc*) gene is important in the early stages of the transformation of the colonic epithelium. The *Apc* gene encodes a tumor suppressor protein that is linked to 80% of cases of sporadic CRC, and which is involved in the inherited condition, familial adenomatous polyposis syndrome (5). Besides the loss of the APC protein and exposure to environmental factors, including cigarette smoke and alcohol (6), a poor diet and high body mass index (BMI) (7) also contribute to the risk of CRC. The mitogen-activated protein kinase (MAPK) pathway is known to transduce these signals, leading to various biological outcomes, including apoptosis, inflammation and tumorigenesis (8). The MAPK family of proteins comprises extracellular signal-regulated kinase 1/2 (ERK1/2), c-Jun N-terminal kinase (JNK) and p38. The p38 subfamily consists of four isoforms (p38α, -β, -γ, and -δ) that are defined by the threonine-glycine-tyrosine (Thr-Gly-Tyr; TGY) dual phosphorylation motif, and exhibit significant homology at the amino acid level. p38α and -β are ubiquitously expressed and are thought to have overlapping functions, while p38γ and -δ are differentially expressed, depending on the tissue type. p38 is activated through phosphorylation at the Thr^180^-Gly-Tyr^182^ motif by MAP kinase kinase 3 (MKK3), MKK4 and MKK6 (9). The activation of p38 results in various cellular changes, including the regulation of transcription, protein synthesis, cell surface receptor expression, cell cycle progression and apoptosis. The oncogenic function of p38 is context-dependent. In CRC, p38α is required for the proliferation and survival of CRC cells, and its inhibition leads to cell cycle arrest and autophagic cell death (10). p38 has been demonstrated to be involved in CRC cell migration and metastasis in animal models (11,12). Furthermore, it has been revealed that the inhibition of p38 results in a loss of CRC cell resistance to chemotherapy drugs (13). Therefore, p38 activity is important in CRC tumorigenesis and resistance to chemotherapy.

Regardless of its importance, few studies have examined the contribution of p38 genetic polymorphisms to the risk of CRC. The present study investigated the functional importance of an SNP located in the promoter region of p38β, and correlated its presence with the risk of sporadic CRC. To the best of our knowledge, the study demonstrates for the first time that the p38β promoter region SNP (rs2235356, -1628A>G) is correlated with an increased risk of sporadic CRC, in a study cohort.

## Materials and methods

### Study subjects and sample collection

The study was approved by the Institutional Review Board of Sun Yat-Sen University (Guangzhou, China). The study cohort consisted of 855 patients with histologically confirmed sporadic CRC and 871 cancer-free control subjects, who were genetically unrelated Han Chinese individuals from Guangzhou and the surrounding regions of Southern China (14). The response rate was 95%. The exclusion criteria removed individuals with familial adenomatous polyposis and those fulfilling the Amsterdam criteria for hereditary nonpolyposis CRC from the study. The 871 cancer-free control subjects were randomly selected from a pool of >10,000 individuals who had participated in the health check-up programs in the community health stations in Guangzhou during the same period of time as when the cases were recruited. The response rate was 85%. The controls were frequency-matched to the cases by gender and age (±5 years). Following the receipt of written informed consent, each participant was scheduled for an interview that used a structured questionnaire to collect information on various factors, including smoking status, alcohol use and the family history of cancer. The definitions of these factors corresponded with those used in a previous study by our group (14). The present study used the BMI cutoff points suggested by the Cooperative Meta-Analysis Group of the Working Group on Obesity in China (15). Subjects whose BMI was <18.0 kg/m^2^ were categorized as being underweight; whereas 18.0–25.0 kg/m^2^ constituted normal body weight and >25.0 kg/m^2^ was classed as overweight. Each subject was requested to donate 5 ml blood.

### Genotyping

**Figure 1.**
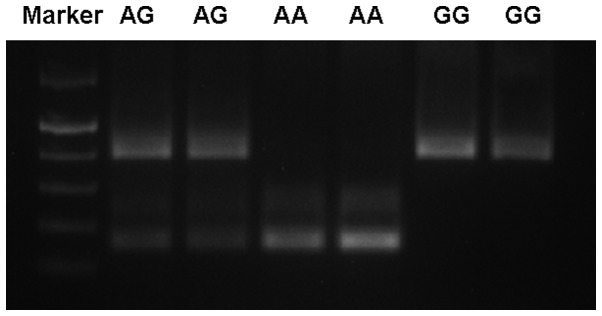
Representative image for rs2235356 genotyping. Polymerase chain reaction (PCR) products were digested by FokI, followed by electrophoresis using 1% agarose gel. Lane 1, DNA ladder; lanes 2 and 3, -1628AG heterozygote genotype; lanes 4 and 5, -1628AA genotype; lanes 6 and 7, -1628GG genotype.

**Figure 2.**
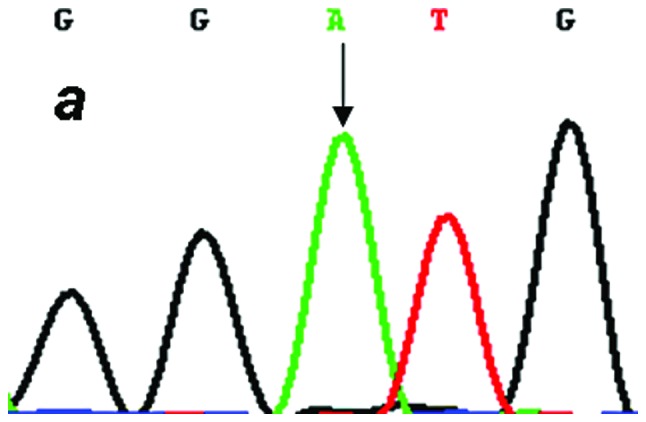
Determining the p38β -1628A>G genotype by DNA sequencing: the -1628AA genotype.

**Figure 3.**
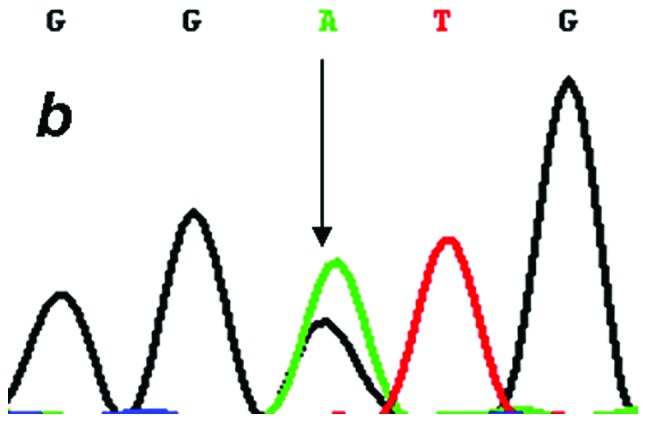
Determining the p38β -1628A>G genotype by DNA sequencing: the -1628AG genotype.

**Figure 4.**
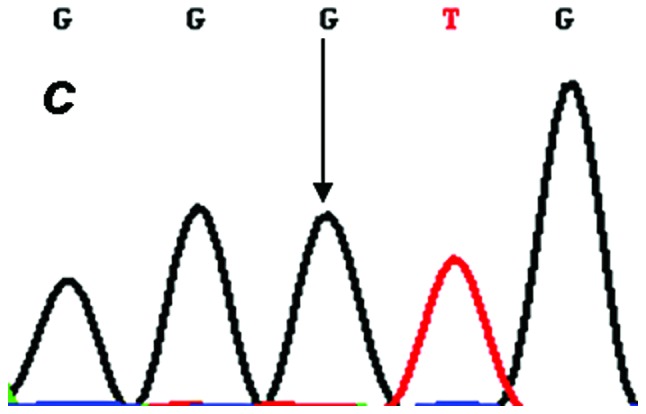
Determining the p38β -1628A>G genotype by DNA sequencing: the -1628GG genotype.

The p38β (rs2235356, -1628A>G) polymorphism was detected by the polymerase chain reaction-restriction fragment length polymorphism (PCR-RFLP) method. In brief, the genomic DNA from the peripheral blood of the participants was extracted by the DNeasy Blood and Tissue kit (Qiagen, Hilden, Germany) and subjected to PCR using the forward primer, 5′-CCGAGGTTTTGTGCAGAGTT-3′, and the reverse primer, 5′-CAGGTCAGCTCAGTGATGAGA-3′. The PCR conditions used were as follows: 94°C for 5 min, 35 cycles of 94°C for 30 sec, 60°C for 45 sec and 72°C for 60 sec, and a final extension step of 72°C for 10 min. The PCR amplification was further digested with FokI (New England Biolabs, Inc., Ipswich, MA, USA) overnight at 37°C, followed by agarose gel electrophoresis to reveal the genotype of either -1628AA (two bands of 157 and 228 bp), -1628GG (one band of 385 bp) or the heterozygous -1628AG (three bands of 157, 228 and 385 bp) (Fig. 1). A GeneRuler Low Ranger DNA Ladder (100 bp; Thermo Scientific, Waltham, MA, USA) was used. Two researchers who were blinded to the CRC status of the participants independently evaluated the gel electrophoresis results. The PCR-RFLP assay was repeated in 10% of the samples, which yielded identical results (data not shown). For each target genotype the PCR products were purified and confirmed by direct sequencing (Figs. 2–4). An *in silico* analysis was performed using Genomatix (Genomatix Software GmbH, Munich, Germany) to identify transcription factors that either acquired or lost the ability to bind to -1628AA in the p38β promoter region due to the SNP.

### Statistical analysis

A two-sided χ^2^ test was used to evaluate the differences in the distributions of age, gender, smoking status, alcohol use, BMI and family history of cancer between the CRC cases and the cancer-free controls. The Hardy-Weinberg equilibrium (HWE), determined by a χ^2^ goodness of fit test, was used to compare the expected and the observed genotype frequencies in the cancer-free controls. Unconditional logistic regression was used to calculate the odds ratios (ORs) and 95% confident intervals (CIs) to estimate the correlation between the presence of the SNP and the CRC risk, with and without adjustments for age, gender, smoking status, alcohol drinking status, BMI and family history of cancer. A logistic regression model was also used for the trend test. In the stratification analysis, the main effects of the p38β polymorphisms were assessed in each subgroup, along with the effect of potential interactions between the p38β polymorphisms and selected variables on cancer risk. All two-sided statistical tests were performed using SAS software version 9.1 (SAS Institute, Inc., Cary, NC, USA). P<0.05 was considered to indicate a statistically significant difference.

## Results

### Characteristics of patients with CRC and the control subjects

The demographic characteristics of the 855 CRC cases and 871 cancer-free controls are presented in Table I. There was no significant difference in the distributions of age (P=0.5773) and gender (P=0.9319) between the two groups. However, the patients with CRC were more likely to consume alcohol (46.8% in CRC cases, compared with 23.4% in the controls; P<0.0001) and, in female participants, be menopausal (80.5% in CRC cases, compared with 57.9% in controls; P<0.0001). Furthermore, there were a greater number of CRC cases with an abnormal BMI or a family history of cancer compared with the controls (P=0.0245 and P=0.0379, respectively). These variables were therefore adjusted in the multivariate logistic regression analysis, as well as in the stratification and gene-environment interaction analysis, to reveal the effect of the studied SNP on the risk of CRC.

### Distribution of the p38β promoter region SNP and the risk of sporadic CRC

The genotypic and allelic distributions of the p38β -1628A>G polymorphism among the cases and controls are summarized in Table II. The observed genotype frequencies of this SNP were in agreement with the Hardy-Weinberg equilibrium in the control subjects (P=0.796). As demonstrated in Table II, the logistic regression analysis indicated that the subjects carrying the -1628G variant allele (-1628AG and -1628GG) had a 1.99-fold increased risk of sporadic CRC compared with those who were homozygous for the -1628A allele (95% CI, 1.6–2.47; P<0.0001). The increase in CRC risk was particularly evident in the subjects who were heterozygous for the -1628G allele, with a 2.22-fold increase in risk (95% CI, 1.78-2.79; P<0.0001). There was a significant trend for the allele dose effect on the risk of CRC (P_trend_<0.0001). The correlation between the -1628G variant allele and the risk of CRC was also statistically significant at a 5% type I error level, having adjusted for multiple tests using a Bonferroni correction (P<0.0001).

### Stratification analyses of the p38β promoter region SNP and the risk of CRC

A stratification analysis of the correlations between the p38β variant genotypes and the risk of sporadic CRC was performed by subdividing the subjects by age, gender, smoking and alcohol consumption statuses, family history of cancer and BMI (Table III). With the exception of the subjects who had a family history of cancer, the subjects carrying the -1628G variant allele (-1628AG and -1628GG) were correlated with an increased risk of CRC in all remaining subgroups (Table III).

### In silico analyses of the affect of the p38β promoter region SNP on p38β gene expression

The presence of the SNP in the p38β promoter region may affect the gene expression of p38β. An *in silico* analysis was performed using Genomatix (Genomatix Software GmbH, Munich, Germany), to identify the transcription factors that had either acquired or lost the ability to bind to this locus due to the SNP. It was determined that the -1628G variant allele was correlated with a loss of binding ability for chorion-specific transcription factor (GCMa), but also with an acquired binding ability for basic Krüppel-like factor (KLF3), erythoid Krüppel-like factor (EKLF), the rat C2H2 zinc finger protein (involved in olfactory neuronal differentiation), Wilms' tumor suppressor, the zinc finger with Krüppel-associated box (KRAB) and SCAN (named after SRE-ZBP, CTfin51, AW-1 and Number 18 cDNA) domains 3 (data not shown).

## Discussion

The present study investigated the correlation between a putative functional SNP of the p38β promoter region (rs2235356) and the risk of CRC in a Chinese population, with a sample size of 855 patients with sporadic CRC and 871 cancer-free control subjects. We developed a PCR-based method for detecting this SNP, and demonstrated that the -1628G allele was correlated with an increased risk of sporadic CRC in our study cohort (95% CI, 1.6–2.47; P<0.0001).

p38 promotes the survival of CRC cells, primarily through its functions in the DNA repair pathway and in autophagy. p38 is one of the effector kinases of the DNA damage sensor system, following the activation of ataxia telangiectasia mutated (ATM) kinase, ataxia telangiectasia and Rad3-related (ATR) kinase and DNA-dependent protein kinase (DNA-PK) (16). Therefore, p38 may enhance the DNA repair response following chemotherapy in colon cancer cells, resulting in drug resistance. Furthermore, p38 has been demonstrated to inhibit the autophagy and cell death of CRC cells (17), indicating that p38 activity is necessary for the survival of these cells. The present study identified an SNP in the p38β promoter region that may be biologically significant in the tumorigenesis of sporadic CRC, which is consistent with the importance of p38 in mediating CRC.

One of the primary downstream effectors of p38 is activator protein-1 (AP-1), a heterodimeric transcription factor composed of proteins belonging to the c-fos, c-Jun and activating transcription factor (ATF) families. In CRC, AP-1 activity is activated by ERK and JNK (18), or by the hyper-activation of the Wnt pathway. p38 phosphorylates ATF-2, which forms a heterodimer with the Jun family transcription factors to form active AP-1 (19). Aberrant c-Jun activity has been observed in CRC, which is consistent with the function of p38 in the regulation of AP-1 (20). Abnormal p38 activity may increase the risk of CRC by modulating AP-1. In addition to JNK and ATF-2, AP-1 activity is also regulated by the level of c-fos. The expression of c-fos is dependent on sterol regulatory element, which interacts with ternary complex factors (TCFs). Sap-1a is a TCF that is phosphorylated by p38, thus AP-1 activity may also be regulated by p38 through Sap-1a. Therefore, aberrant levels of the p38 pathway constituents may promote CRC through the activation of the AP-1 transcription factor.

In conclusion, the present study has provided evidence that the -1628A>G genetic variation in the p38β promoter region may contribute to the susceptibility to CRC in Chinese populations. It would be of interest to determine if this SNP is correlated with the risk of sporadic CRC in other ethnic groups. Furthermore, future efforts are required to determine the functional importance of this SNP in controlling the level of p38β.

## Figures and Tables

**Table I.** Frequency distributions of selected variables in patients with CRC and cancer-free control subjects.

Variables	No. cases (n=855) (%)	No. controls (n=871) (%)	P-value
Age (years)			0.5773
≤49	198 (23.1)	198 (22.7)	
50-60	234 (27.4)	258 (29.6)	
>60	423 (49.5)	415 (47.7)	
Gender			0.9319
Male	521 (60.9)	529 (60.7)	
Female	334 (39.1)	342 (39.3)	
Smoking status			0.4520
Smoker	414 (48.4)	406 (46.6)	
Non-smoker	441 (51.6)	465 (53.4)	
Alcohol status			<0.0001
Not teetotal	400 (46.8)	204 (23.4)	
Teetotal	455 (53.2)	667 (76.6)	
Family history of cancer			0.0379
Yes	96 (11.2)	72 (8.3)	
No	759 (88.8)	799 (91.7)	
BMI (kg/m^2^)			0.0245
<18	56 (6.5)	35 (4.0)	
18–25	557 (65.2)	608 (69.8)	
>25	242 (28.3)	228 (26.2)	
Menstrual history			<0.0001
Premenopause	65 (19.5)	144 (42.1)	
Menopause	269 (80.5)	198 (57.9)	

P-value for a two-sided χ^2^ test. BMI, body mass index; CRC, colorectal cancer.

**Table II.** p38β promoter region polymorphism (rs2235356, -1628A>G) is correlated with an increased risk of CRC.

	No. cases (%)	No. controls^a^ (%)	P-value^b^	Crude OR (95% CI)	Adjusted OR^c^ (95% CI)
Total no. subjects	855	871			
Total no. alleles	1710	1742			
Rs2235356A>G genotype			<.0001		
AA	214 (25.0)	343 (39.4)		1.00 (ref.)	1.00 (ref.)
AG	551 (64.5)	410 (47.1)		2.15 (1.74–2.76)	2.22 (1.78–2.79)
GG	90 (10.5)	118 (13.5)		1.22 (0.89–1.69)	1.21 (0.86–1.70)
AG+GG	641 (75.0)	528 (60.6)		1.95 (1.58–2.39)	1.99 (1.60–2.47)
Trend test P-value				0.0002	0.0004
G allele	0.427	0.371	<.0001		

a Observed genotype frequencies among the control subjects were in agreement with Hardy-Weinberg equilibrium [p^2^+2pq+q^2^=1, where p represents the frequency of allele (A) and q represents the frequency of allele (G)] (χ^2^=0.067 and P= 0.796 for rs2235356A>G);

b two-sided χ^2^ test to demonstrate the differences in the distribution of genotype frequencies between cases and controls;

c adjusted in a logistic regression model that included age, gender, smoking status, alcohol use, body mass index and family history of cancer. OR, odds ratio; CI, confidence interval; CRC, colorectal cancer.

**Table III.** Stratification analysis of the p38β promoter region polymorphism (rs2235356, -1628A>G) genotypes by selected variables in patients with CRC and cancer-free control subjects.

Variable	No. cases (n=855) (%)		No. controls (n=871) (%)		Crude OR (95% CI) AG+GG vs. AA	Adjusted OR^a^ (95% CI) AG+GG vs. AA	P-value^b^
	AA	AG+GG	AA	AG+GG			
Age (years)							0.2772
≤60	103 (23.8)	329 (76.2)	182 (39.9)	274 (60.1)	2.12 (1.59–2.84)	2.11 (1.56–2.87)	
> 60	111 (26.2)	312 (73.8)	161 (38.8)	254 (61.2)	1.78 (1.33–2.39)	1.97 (1.43–2.72)	
Gender							0.2622
Male	135 (25.9)	386 (74.1)	204 (38.6)	325 (61.4)	1.80 (1.38–2.33)	1.82 (1.36–2.44)	
Female	79 (23.7)	255 (76.3)	139 (40.6)	203 (59.4)	2.21 (1.59–3.08)	2.10 (1.50–2.94)	
Smoking status							0.0770
Smoker	112 (27.1)	302 (72.9)	154 (37.9)	252 (62.1)	1.65 (1.23–2.21)	1.68 (1.20–2.35)	
Non-smoker	102 (23.1)	339 (76.9)	189 (40.6)	276 (59.4)	2.28 (1.71–3.04)	2.22 (1.66–2.97)	
Drinking status							0.0501
Not teetotal	114 (28.5)	286 (71.5)	76 (37.3)	128 (62.7)	1.49 (1.04–2.13)	1.47 (1.02–2.12)	
Teetotal	100 (22.0)	355 (78.0)	267 (40.0)	400 (60.0)	2.37 (1.81–3.11)	2.37 (1.79–3.14)	
BMI (kg/m^2^)							0.3030
<18	9 (16.1)	47 (83.9)	12 (34.3)	23 (65.7)	2.72 (1.01–7.39)	3.63 (1.01–13.0)	
18–25	129 (23.2)	428 (76.8)	231 (38.0)	377 (62.0)	2.03 (1.57–2.63)	2.01 (1.53–2.62)	
>25	76 (31.4)	166 (68.6)	100 (43.9)	128 (56.1)	1.71 (1.17–2.49)	1.94 (1.29–2.92)	
Family history of cancer							0.0578
Yes	23 (24.0)	73 (76.0)	19 (26.4)	53 (73.6)	1.14 (0.56–2.30)	1.04 (0.49–2.21)	
No	191 (25.2)	568 (74.8)	324 (40.5)	475 (59.5)	2.03 (1.63–2.52)	2.12 (1.69–2.66)	

a Adjusted for age, gender, smoking status, alcohol use, body mass index (BMI) and family history of cancer in a logistic regression model;

b calculated using a standard unconditional logistic regression model for the multiplicative interactions between the rs2235356 1628A>G polymorphism and selected variables. OR, odds ratio; CI, confidence interval; CRC, colorectal cancer.
